# Cats as Carriers of Disease

**Published:** 1917-02-17

**Authors:** 


### CATS AS CARRIERS OF DISEASE.

### Inflammation of the Eye from Infected Fur.

At a recent meeting of the Section of Ophthalmology of the Royal Society of Medicine a paper was read by Mr. Arnold Lawson on three cases of conjunctival infection traced to the infected fur of cats which the patients were in the habit of fondling. The case histories are opportune because it is extremely difficult to persuade most people, and especially those who are accustomed to constant handling of pet cats or pet lapdogs, that there is a real risk of health involved by too close a personal contact with their favourite animals. Mr. Lawson has therefore done good service in bringing forward these three cases, which all occurred in the children of well-to-do parents. Not that children alone show disregard (or ignorance) of the laws of health in this respect; adults, too, are careless of hygiene in just the same way, though less frequently than children.

### A Plea for Fuller Investigation.

A comprehensive investigation of the diseases that are transferable from domestic animals to man is one of those measures of importance from a public health point of view which would well repay the labour involved. During the war there is perhaps not much chance of the Local Government Board or the Medical Research Committee putting their hands to the task, because there are greater problems of more urgency waiting to be tackled. The subject, however, will have to be threshed out some day; and contributions to it such as that of Mr. Lawson in the cases described below are useful in that they demonstrate the actual sequence of the spread of infection from animal to human being, and are thus not liable to dismissal as mere surmises or prejudiced generalisations.

That cats can convey the infection of diphtheria and may start serious epidemics of that disease has long been strongly suspected, one might almost say proved: this point is one deserving of further research, to establish the facts one way or the other. That ringworm, actinomycosis, and other disorders can also be contracted from association with animals is highly probable. The subjoined cases show that streptococcal and staphylococcal conjunctivitis and tuberculosis must be added to the list; and support the case outlined above for a really thorough probing of the whole subject.

### Our National Contempt of Science.

During the last few years there has been a great development of the habit of fondling cats, lapdogs, monkeys, and other animal pets, chiefly among spoilt women of the plutocracy and the *demi-monde*. That these customs are insanitary and to a slight but quite definite degree dangerous we do not hesitate to affirm. The medical profession knows this quite well; and has done a good deal, if not all that it might have done, to point out the fact to the public at large. That the lesson has not been accepted is due partly, perhaps, to insufficient zeal on the part of the preachers of hygiene; but chiefly, in our opinion, to that profound contempt for all scientific learning which characterises the product of our British system of education.

### Three Cases of Fur Infection of the Human Conjunctiva from Cats.

The details of Mr. Lawson's cases are as follows:—

*Case* 1.—A girl of fifteen, an only child, was much devoted to a Persian cat, which she frequently fondled. One evening, after burying her face in the cat's fur, her eyes became suffused and she had a severe sneezing fit. On the following day the eyes were swollen and a discharge exuded, the condition becoming very acute by evening. The nostrils were also swollen and purulent, and sanguineous matter freely oozed therefrom. *Staphylococcus pyogenes aureus* was cultivated in abundance from both the discharge and the cat's fur. After a few anxious days improvement ensued, and recovery was complete without permanent damage to the eyes. The cat was destroyed.

*Case* 2.—A boy who, a fortnight before being seen by Mr. Lawson, had slight inflammation in the left eye, with some discharge and swelling of the submaxillary glands, which the local doctors diagnosed as mumps. But it did not run the usual course of mumps, and, as the condition became worse Mr. Lawson was consulted. During this time there had been neither fever nor pain. The whole of the left side of the face was swollen, and a brawny induration was present over the angle of the jaw, and extended to the neck. The lids of the left eye were so swollen that examination was difficult. There was a muco-purulent discharge in the furrows of the swollen tissues. Tubercle seemed unlikely, on account of the rapid development of the conditions. The boy had been in the habit of walking about with his Persian cat perched on his left shoulder; he also cuddled it. Under an anæsthetic, vigorous scraping and swabbing was performed, including the application of a 2 per cent. solution of silver nitrate. The glandular swelling began to subside, but a complete cure was not accomplished until twelve weeks. The cultivation of the scrapings produced *Streptococcus longus*, *Streptococcus brevis*, and *Staphylococcus albus*. The measures were directed to dealing with the streptococci. Similar organisms were found in the cat.

*Case* 3.—A patient of five, a little girl. While staying with relatives in the country, adjacent to a farm, she had muco-purulent conjunctivitis, of mild degree, which had come on shortly after nursing a cat which was a great favourite, and which divided its attention between the child and the denizens of a neighbouring cow-shed. After a time a number of neck glands were involved, and this aspect of the condition became serious. On the upper lid he found an exuberant mass of cock's-comb granulations. He suspected it was a case of conjunctival tubercle. He cut away and scraped the infected tissues, finishing with the silver nitrate stick. An excellent recovery eventuated. Mr. Browning's report on the scrapings was that the disease was tubercle, probably of the bovine variety. Inoculation of very few of the bacilli into a guinea-pig caused it to die of acute tubercle within a fortnight.

Mr. Lawson explained that he had brought the cases forward because the general public do not yet realise the great danger of cats as carriers of disease, especially among children. The constant habit of licking themselves in which cats indulge (thereby distributing their saliva) has led to the view that they are cleanly animals.

